# Chemical chaperone TUDCA prevents apoptosis and improves survival during polymicrobial sepsis in mice

**DOI:** 10.1038/srep34702

**Published:** 2016-10-03

**Authors:** Marcel Doerflinger, Jason Glab, Christina Nedeva, Irvin Jose, Ann Lin, Lorraine O’Reilly, Cody Allison, Marc Pellegrini, Richard S. Hotchkiss, Hamsa Puthalakath

**Affiliations:** 1 Department of Biochemistry and Genetics, La Trobe Institute of Molecular Science, La Trobe University, Kingsbury Dr. Victoria 3086, Australia; 2 The Walter and Eliza Hall Institute of Medical Research, Parkville, VIC 3052, Australia; 3 Department of Medical Biology, University of Melbourne, Parkville, VIC 3050, Australia; 4 School of Medicine, Department of Anesthesiology, Washington University, 660 South Euclid, St. Louis, MO 63110, USA

## Abstract

Sepsis-induced lymphopenia is a major cause of morbidities in intensive care units and in populations with chronic conditions such as renal failure, diabetes, HIV and alcohol abuse. Currently, other than supportive care and antibiotics, there are no treatments for this condition. We developed an *in vitro* assay to understand the role of the ER-stress-mediated apoptosis process in lymphocyte death during polymicrobial sepsis, which was reproducible in *in vivo* mouse models. Modulating ER stress using chemical chaperones significantly reduced the induction of the pro-apoptotic protein Bim both i*n vitro* and in mice. Furthermore, in a ‘two-hit’ pneumonia model in mice, we have been able to demonstrate that administration of the chemical chaperone TUDCA helped to maintain lymphocyte homeostasis by significantly reducing lymphocyte apoptosis and this correlated with four-fold improvement in survival. Our results demonstrate a novel therapeutic opportunity for treating sepsis-induced lymphopenia in humans.

In the United States alone, ∼750000 individuals develop sepsis (or colloquially known as blood poisoning), and of these ∼30% succumb to this disorder annually. Sepsis is the 10^th^ leading cause of death, with an enormous financial burden (sepsis costs between US$25,000 and US$50,000 per episode^1^). Though better treatment methods have improved overall patient survival, sepsis is still a major health and economic strain due to an ageing population^2^. Sepsis is defined as the host inflammatory response to severe, life-threatening infection with the presence of organ dysfunction. The host immune response to sepsis can be divided into two stages, a hyper-inflammatory phase and a hypo-inflammatory phase. During the hyper-inflammatory phase, activated immune cells (mostly the innate immune system) produce copious amounts of inflammatory cytokines, which can result in multiple organ failure. However, improved treatment protocols have resulted in most patients surviving this stage and entering a protracted immune suppressive phase^3^. The latter phase is characterized by extensive apoptosis in the cells of the adaptive immune system, i.e., B cells, and T cells^2^ leading to prolonged lymphopenia. The severe lymphopenia and other accompanying immune defects render patients unable to clear their primary infection and susceptible to lethal nosocomial infections.

Experimental drug therapies for sepsis are currently at a crossroad with more than 30 drug trials failing in the last 25 years. These include, but not limited to, *Eritoran* or anti-TLR4 compound (Eisai Co. Ltd, Japan), *Xigris* or activated protein C (Eli Lilly & Co. USA), *CytoFab* or anti-TNFα antibody (AstraZeneca, Sweden) and *Talactoferrin alfa,* an immuno-modulatory lactoferrin (Agennix, Germany) to name a few. Failure of these trials of different anti-inflammatory agents highlights the fact that inflammation is not the key driving mechanism of sepsis-related fatalities. There is an inverse correlation between immune cell apoptosis and patient survival i.e. lymphocyte apoptosis is the major reason for most fatalities associated with sepsis^3^,^4^. The pro-apoptotic protein Bim is considered to be an essential initiator of apoptosis in a wide variety of physiological settings and especially in lymphocyte homeostasis^5^. While the role of Bim-mediated lymphocyte apoptosis in sepsis is well documented^6^,^7^ and *Bim* is induced to very high levels in lymphocytes from patients with early stage severe sepsis^8^, the exact mechanism of this cell death is unknown. We have previously shown that ER-stress mediated apoptosis is regulated by Bim in numerous cell types including lymphocytes^9^.

In the present study, we demonstrate that ER-stress during sepsis induces Bim-mediated lymphocyte death. Modulating endoplasmic stress (ER stress) by chemical chaperones significantly reduces Bim up-regulation and improves survival in a mouse two-hit sepsis model. Our study defines a novel therapeutic strategy for treating sepsis with Gram-negative bacterial infections.

## Results

### Development of an *in vitro* system to study role of ER stress in lymphopenia

**Figure 1:**
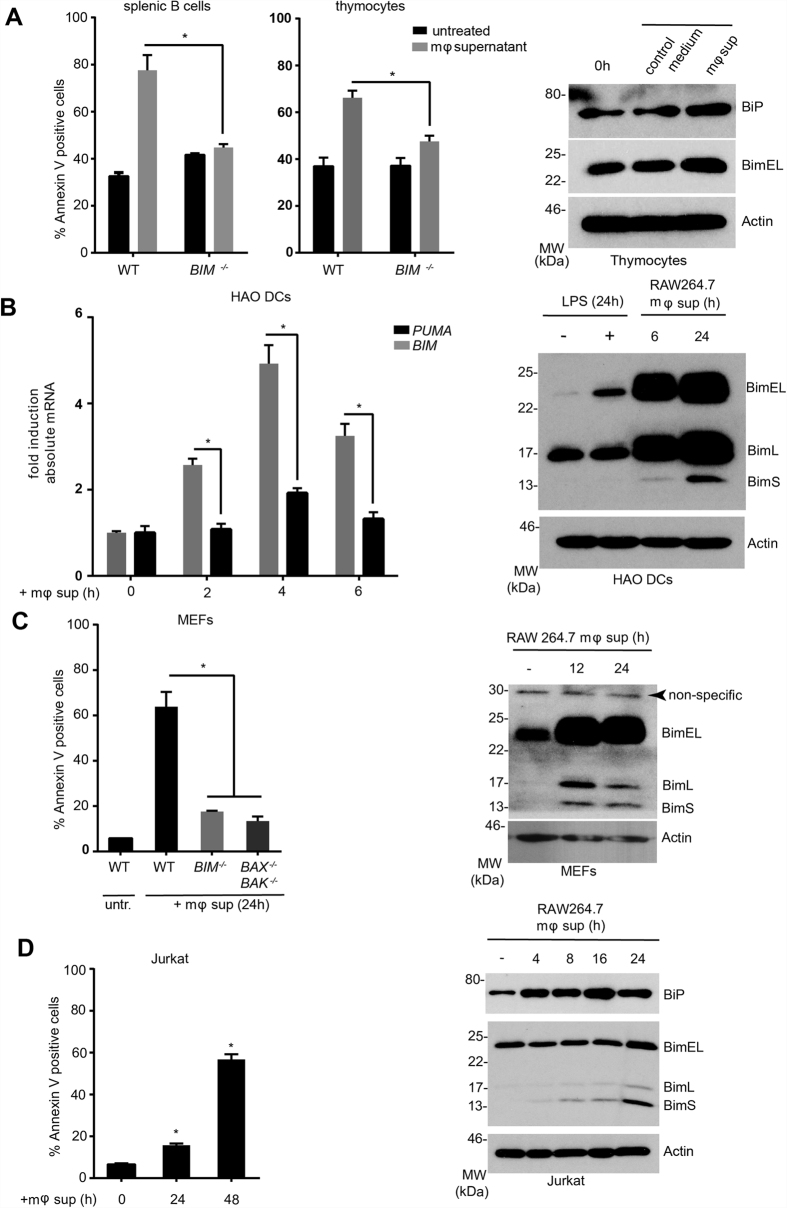
Macrophage conditioned medium induces apoptosis and Bim induction *in vitro*. RAW264.7 cells were activated with LPS (100 ng/ml) for 24 hours and the medium was used for treating target cells. In each case, apoptosis was measured by annexin V-PI staining. For protein analysis by Western blots and mRNA analysis by droplet digital PCR. (**A**) Mouse thymocytes and splenic B cells from WT or *BIM*^*−/−*^ mice were treated with the conditioned medium and apoptosis was measured after 24 hours. Thymocytes treated with the conditioned medium were analysed by Western blot for BiP and Bim induction after 6 hours (right). (**B**) HAO cells were treated as in (**A**) and *BIM* and *PUMA* mRNA (left) and protein induction (right) were measured at the indicated time points. (**C**) MEFs from WT, *BIM*^*−/−*^ and *BAX*^*−/−*^*/BAK*^*−/−*^ mice were treated as in (**A**) and apoptosis (left) and Bim protein induction (right) were analysed. (**D**) Jurkat T cells were treated for indicated time points (h) and apoptosis and Bim protein induction were analysed. Error bars +/− SEM; n = 3; unpaired, two-tailed Student’s *t* - test. *p < 0.05.

**Figure 2:**
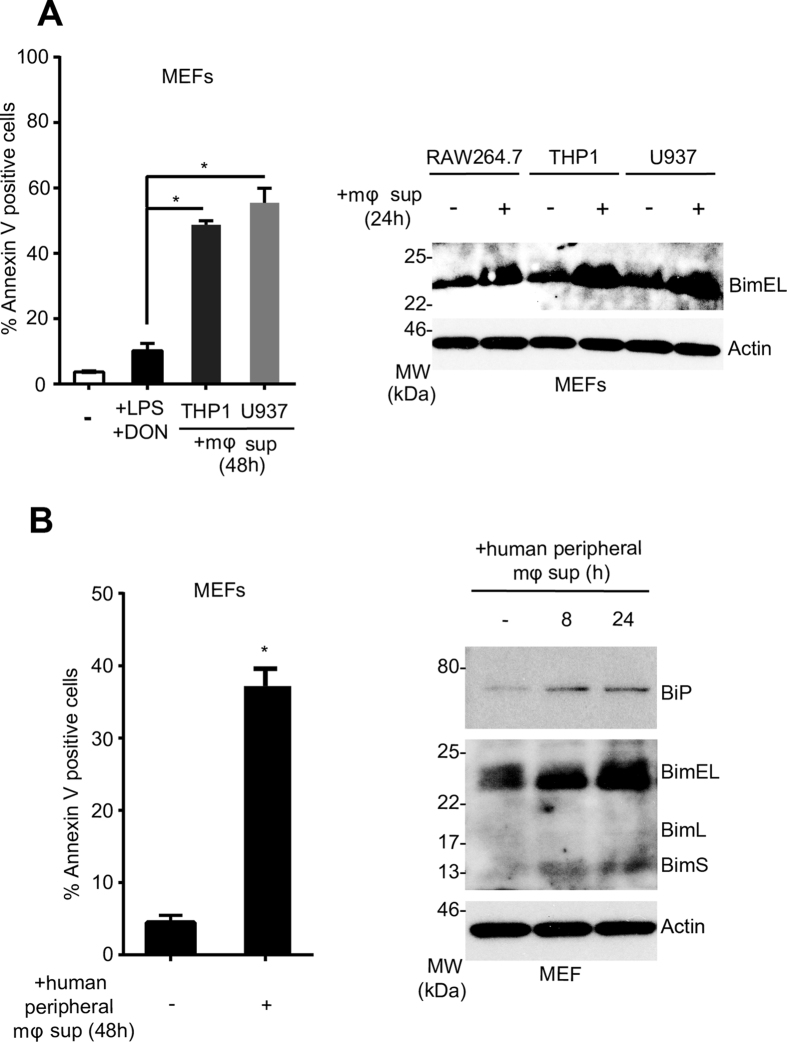
Conditioned media from activated human macrophage cell lines and primary cells induce Bim-mediated apoptosis in target cells. **(A**) THP-1 and U937 cells (human macrophage cell lines) were activated with LPS (100 ng/mL) and deoxynivalenol (DON) (200 ng/mL) and the conditioned medium was collected. The target cells (MEFs) were treated with this conditioned medium and apoptosis was measured after 48 h by annexin V-PI staining (left) or Bim induction was analysed by Western blot. Conditioned medium from activated RAW264.7 cells was used as positive control. (**B**) Human peripheral blood monocytes were differentiated into macrophages and were treated with LPS and DON. The conditioned medium was used to treat the target cells (MEFs). ER stress protein BiP and Bim induction was analysed by Western blot and apoptosis was measured by annexin V-PI staining after 48 h. Error bars +/− SEM; n = 3; unpaired, two-tailed Student’s *t* - test. *p < 0.005.

**Figure 3:**
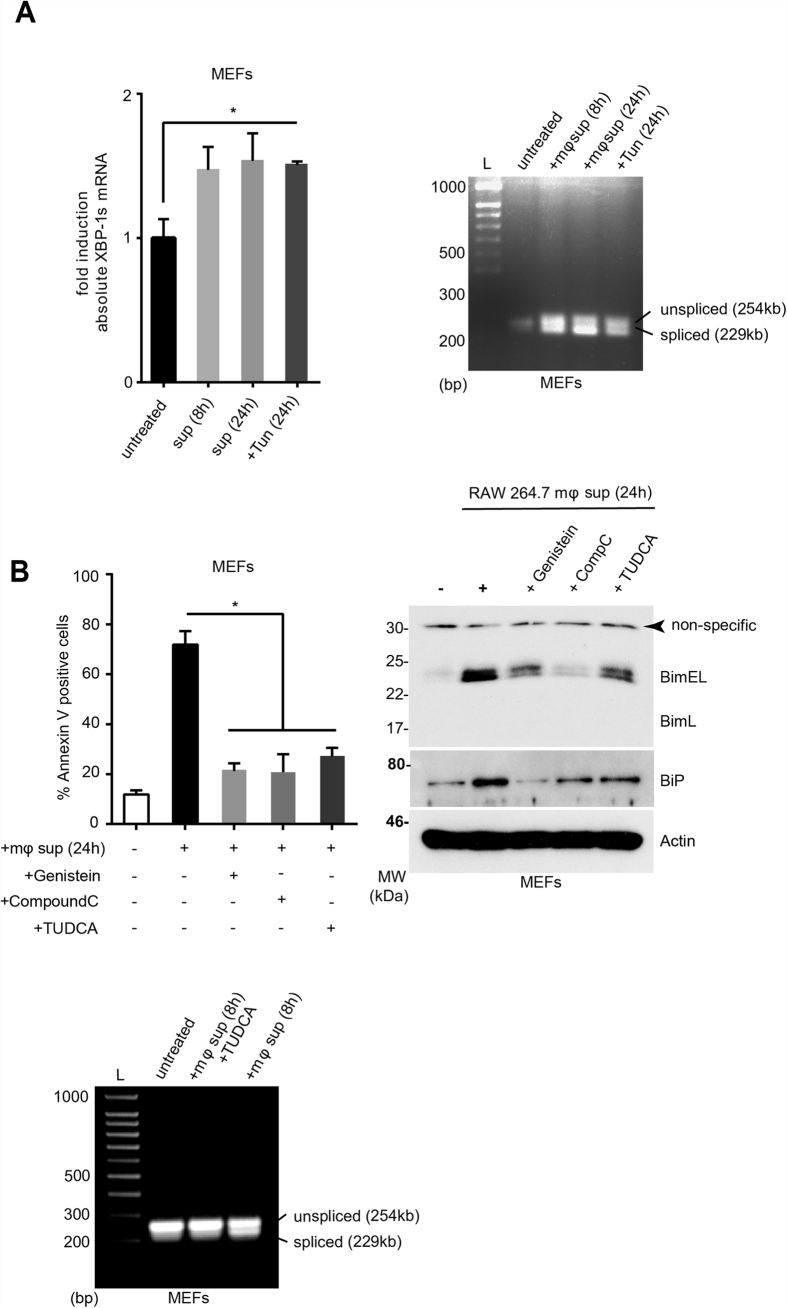
Conditioned medium from activated macrophage cell lines induces ER stress target cells and this can be alleviated by chemical chaperones. **(A**) MEFs were treated with the conditioned medium and *XBP1* splicing was analysed at the indicated time points by digital droplet PCR (left) or by conventional PCR (right). Tunicamycin (200 ng/ml) was used as positive control. (**B**) MEFs were treated with the conditioned medium in the absence or in the presence of chemical chaperones TUDCA (1 mg/mL), compound C (1 μM), Genistein (5 μM) and the apoptosis response after 24 h was measured by annexin V-PI staining (left) and Bim/BiP protein modulation was assessed by Western blot (right). *XBP1* splicing in MEFs treated with conditioned medium and TUDCA was assessed by PCR (bottom). The spliced and unspliced forms of Xbp-1 are indicated. Error bars +/− SEM; n = 3; unpaired, two-tailed Student’s *t* - test. *p < 0.05.

Apoptosis of lymphocytes and antigen presenting cells is considered to be a hallmark of the immune suppressive phase of sepsis and correlates with patient death^2^,^4^. Macrophages play a key role in sepsis-mediated apoptosis, as they are both the sentinels and the first line of defense against infection and can modulate the host immune response as producers of a wide range of pro/anti-inflammatory cytokines and chemokines^10^. Pertinently, it has been reported that the serum derived from sepsis patients contains a circulating factor capable of inducing apoptosis in hematopoietic cells *in vitro*^11^. To understand the kinetics of gene regulation both at protein and mRNA levels and to study the effect of chemical chaperones, we developed an *in vitro* sepsis assay. In this assay, we activated the murine macrophage cell line RAW264.7 *or in vitro* differentiated human macrophages or macrophage cell lines with bacterial lipopolysaccharide (LPS; 100 ng/ml). The conditioned medium 24-hours post activation was harvested and the target cells (mouse primary thymocytes and splenic B cells, HAO dendritic cells, embryonic fibroblasts and human Jurkat T cells) were treated with this supernatant to assess their Bim induction and apoptotic response. As shown in Fig. 1A,C, splenic B cells, thymocytes and mouse embryonic fibroblasts (MEFs) showed a Bim-dependent apoptosis. This apoptosis response is accompanied by induction of Bim at both protein and transcript levels (Fig. 1B–D). In addition to murine macrophages, we tested the ability of the human macrophage cell lines (THP1 and U937) and human peripheral blood monocytes-derived macrophages to induce Bim in target cells (MEFs). In all cell lines tested, a reproducible Bim induction was observed (Fig. 2A,B). Bim induction in MEFs and Jurkat cells as well as primary thymocytes was accompanied by the up-regulation of ER stress markers including the ER chaperone BiP (Figs 1C,D and 2B) and spliced *XBP1* (Fig. 3A). This upregulation appeared to be modulated by structurally dissimilar chemical chaperones with UPR modulating ability such as Genistein^12^, compound c/dorsomorphin^13^ and Tauroursodeoxycholic acid (TUDCA)^14^ (Fig. 3B). This suggested that up-regulation of Bim during sepsis could be mediated by the ER-stress response.

### Development of an *in vivo* system for sepsis and lymphopenia

**Figure 4:**
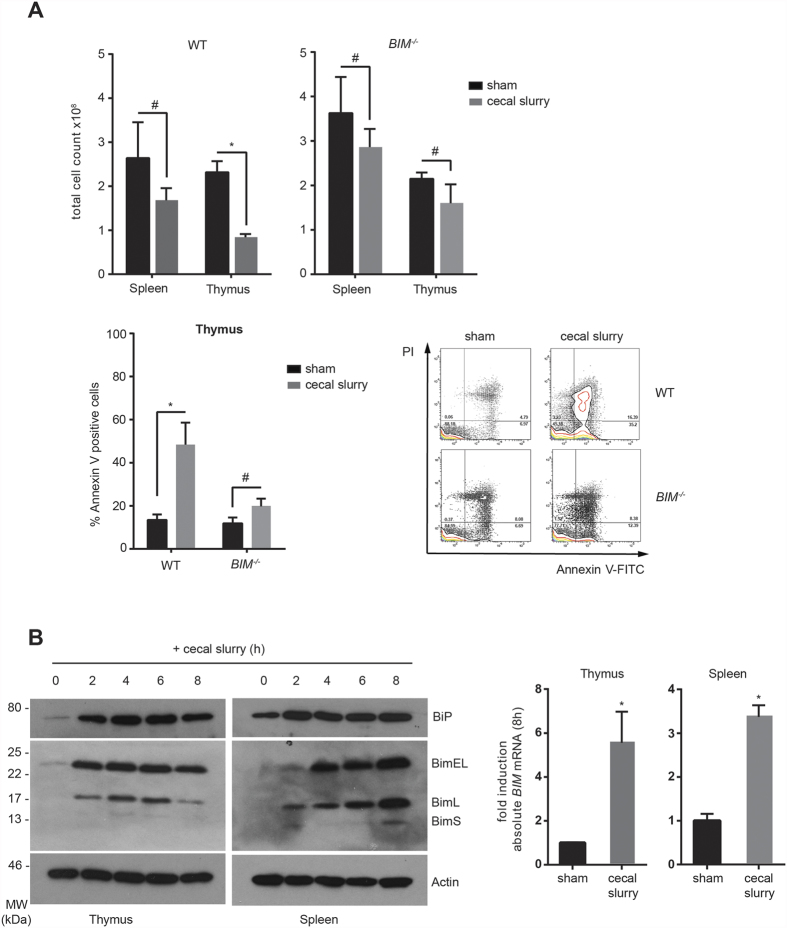
Polymicrobial sepsis in mice elicits Bim-dependent apoptosis. Polymicrobial sepsis was induced by cecal slurry injection in WT and *BIM*^*−/−*^ mice. (**A**) 24 hours post-injection, total cellularity and apoptosis were measured by FACS analysis. (**B**) At the indicated time points after the cecal slurry injection, thymic and splenic tissue was harvested and analysed for Bim protein induction by Western blot (left) or *BIM* mRNA induction by digital PCR analysis (right). Error bars +/− SEM; n = 3; unpaired, two-tailed Student’s *t* - test. *p < 0.05 and ^#^statistically non-significant.

One of the most widely used sepsis models is intravenous/intraperitoneal injection of lipopolysaccharide (LPS) in mice^15^. This is based on the fact that in the majority of sepsis cases, Gram-negative bacteria are involved in the pathogenesis. However, in contrast to the responses observed following bacterial infections, LPS infusion models often do not mimic the changes observed during sepsis^16^. Moreover, the cytokine induction seen after LPS injection is a pre-requirement for the activation of the adaptive immune system^17^, though in a minority of cases, the resulting cytokine storm could lead to fatal organ failure^18^. This led to the development of a more appropriate model i.e. Cecal Ligation and Puncture (CLP), which mimics the clinical course of intra-abdominal sepsis^19^. CLP is considered to be the gold-standard model for sepsis, however, it is a highly invasive procedure that requires extensive training and severity and symptoms can vary significantly depending on the position of the ligation^20^. For our experiments, we followed a far simpler procedure with highly reproducible results where mice were injected (i.p) with cecal slurry (CS) prepared in 5% dextrose^21^. In this model, varying the amount of cecal slurry injected into the mice i.e. between 0.5 g–2.0 g/kg bodyweight, allows us to modulate the severity of disease in a reproducible fashion. Next, we wanted to test if both the apoptotic response and Bim induction in this setting were similar to that which has been reported for the CLP-based sepsis model^6^. As shown in Fig. 4A,B, the apoptotic response of thymocytes and splenocytes was similar previously published reports^6^. Injection of cecal slurry elicited a Bim-dependent apoptotic response i.e. there was a significant reduction in apoptosis in *BIM*^*−/−*^ mice compared with WT mice. These findings were further confirmed by Western blot analysis of Bim protein in the spleen and thymus, in addition to absolute quantification of *BIM* transcripts by digital PCR analysis (Fig. 4B). Taken together, these results suggest that the cecal slurry injection model agrees well with the CLP model both in terms of apoptotic response and Bim induction kinetics.

### ER stress induces Bim during sepsis

Although multiple BH3-only proteins can be induced during ER stress-induced apoptosis^9^, Bim is the major regulator of lymphocyte homeostasis^22^. Additionally in CLP models, ER-stress is known to contribute to abnormal lymphocyte apoptosis^23^. We therefore wanted to test if lymphocytes from mice undergoing sepsis in the cecal slurry injection model were under ER stress and whether modulation of ER stress would reduce Bim induction levels and apoptosis as we observed in our *in vitro* model.

**Figure 5:**
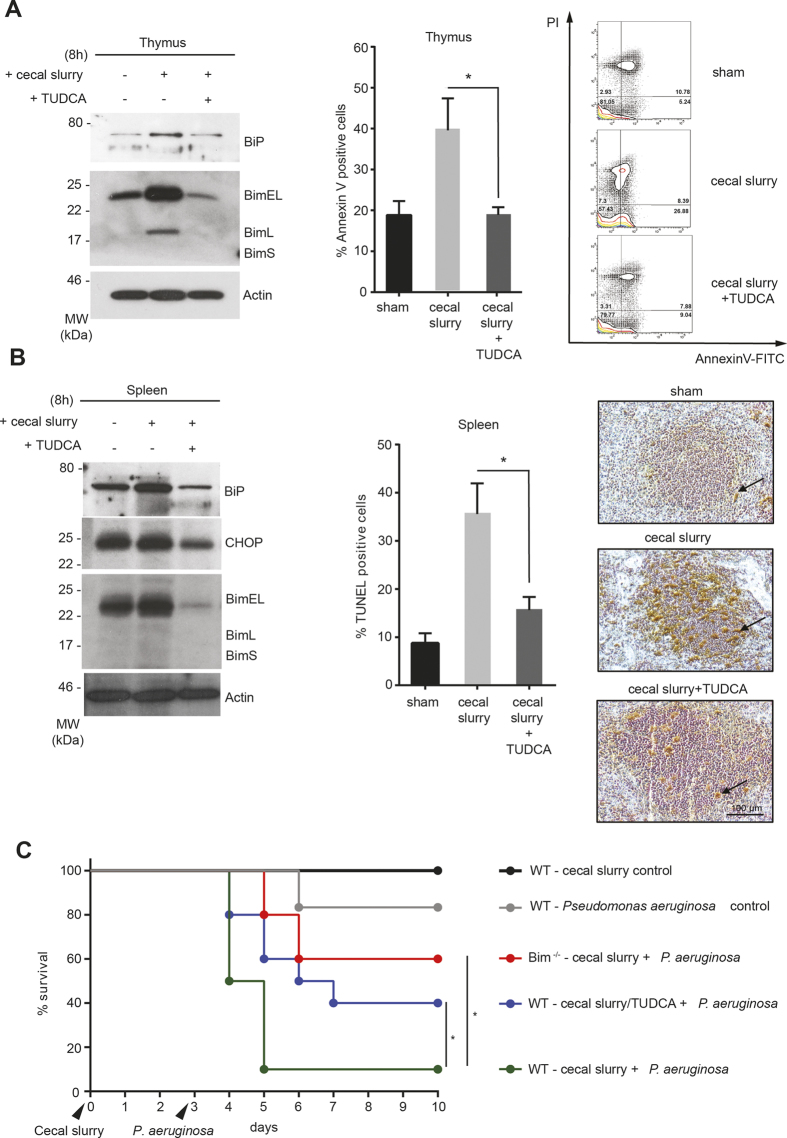
TUDCA treatment significantly reduces ER stress, and improves survival during polymicrobial sepsis. (**A**) Mice were injected with cecal slurry with or without TUDCA and thymic samples were analysed at the indicated time point for ER stress marker protein BiP and Bim (left) and 24 hour later for apoptosis by annexin V-PI staining (right). (**B**) Splenic samples from septic mice were harvested after 8 h and analysed for ER stress markers (left), apoptosis induction was assessed by TUNEL staining after 24 h (right). The arrowheads indicate TUNEL positive cells. The graph in the middle is quantitation of TUNEL positive cells. (**C**) Mice were sham injected or with cecal slurry +/− TUDCA and allowed to recover. On day 3, intra-nasal inoculation of *P. aeruginosa* was performed and survival was measured. Error bars +/− SEM (A) or +/− SD (**B**); n = 3; unpaired, two-tailed Student’s *t* - test. *p < 0.005. (**C**) Log-rank test, n = 5 except for WT with cecal slurry/Pseudomonas +/− TUDCA, where n = 10.

We found that, cecal slurry injection did indeed result in a significant induction of the ER chaperone BiP, (Figs 4B and 5A,B) accompanied by increased expression of the transcription factor Chop, two known markers of ER stress^24^, in thymocytes and splenocytes (Fig. 5B). This was associated with both induction in Bim and apoptosis. However, treating these mice with the chemical chaperone TUDCA resulted in a significant reduction in the levels of the ER stress markers and Bim, suggesting that ER stress could be contributing to the up-regulation of Bim during sepsis. Furthermore, TUDCA treatment resulted in a significant reduction in lymphocyte apoptosis, assessed by both flow cytometry and TUNEL staining of splenic sections (Fig. 5A,B). Taken together, these results suggest that alleviating ER-stress induction with chemical chaperones could be a potential therapeutic strategy for treating sepsis.

### Chemical chaperone administration improves survival in mice in a two-hit pneumonia model

**Figure 6:**
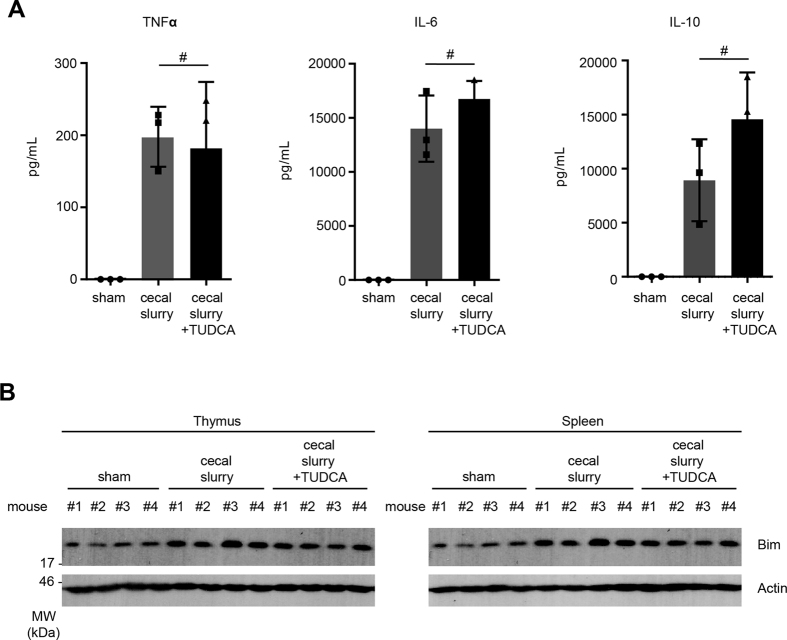
TUDCA treatment does not impair cytokine secretion during polymicrobial sepsis. (**A**) Mice were injected with cecal slurry and serum cytokine levels 6 h post-injection were measured by cytokine bead array. (**B**) Bim protein levels in these mice were analysed by Western blot. Error bars +/− SEM, n = 3; unpaired, two-tailed Student’s *t* - test, *p < 0.005 and ^#^statistically non-significant.

In the majority of currently utilized animal models of sepsis, mortality occurs within the first 3 days i.e. before the animals elicit the adaptive immune response. In contrast, most patients die of sepsis after 3 days of infection due to immune paralysis leading to ventilator-associated pneumonia. To address this apparent disparity, the Hotchkiss group have developed a clinically relevant “two-hit” model of sepsis where mice are challenged with *Pseudomonas aeruginosa*, an opportunistic pathogen, after the onset of immune suppression^25^. We adapted this “two-hit” model to test the efficacy of ER-stress alleviation with the chemical chaperone TUDCA. Our results show that when mice were injected with either PBS or with cecal slurry followed by TUDCA, allowed to recover and then challenged intra-nasally with *P. aeruginosa*, the mice injected with cecal slurry were more susceptible to *P. aeruginosa* compared to sham injected mice (Fig. 5C). Administration of TUDCA post cecal slurry injection significantly improved the survival rate i.e. a 4-fold survival advantage compared to cecal slurry only. This survival advantage could not be attributed to differences in pro-inflammatory cytokine secretion, since TUDCA injection did not lead to any significant difference in the serum cytokine levels between cecal slurry alone or cecal slurry plus TUDCA injected mice, whereas significant reduction in thymic and splenic Bim levels in these mice was indeed noted (Fig. 6A,B).

## Discussion

Our present results corroborate our earlier finding that Bim is a mediator of cellular homeostasis during ER stress-mediated apoptosis^9^. Furthermore, we have provided convincing evidence that ER stress is the main instigator of Bim induction and is associated with lymphocyte apoptosis during sepsis. TUDCA is a hydrophilic bile acid that is produced endogenously in humans at very low levels and has been shown to act as a chemical chaperone that decreases ER stress and UPR signaling and protects hepatocytes and pancreatic islets from ER stress-induced cytotoxicity^14^,^26^. Therefore, we decided to use this and other structurally dissimilar chemical chaperones in our *in vitro* system and demonstrated that this could block Bim induction. We also have demonstrated the practicality of blocking ER stress during sepsis using a clinically relevant model using TUDCA, which clearly offers a survival advantage in mice. Though these studies are conducted in mice, these findings are translationally relevant to humans given the conservation of the mitochondrial apoptotic gene regulation between these two species^27^. We have also provided data using human macrophage cell lines and primary human macrophages as well as human target cells showing a similar apoptotic effect. Furthermore, bile acid derivatives such as chenodeoxycholic (CDCA) and ursodeoxycholic (UDCA) acid have been used in the past as a cholesterol gallstone dissolving agent and in the treatment of chronic cholestatic liver disease^28^ and the tauryl-derivative of deoxycholicacid (TUDCA) has been shown to be more bioavailable and has been approved for treating cirrhosis and gallstones in many countries^29^. TUDCA is also being used in a phase 2 clinical trial for treating juvenile diabetes (NCT02218619), has been shown to improve insulin sensitivity in obese people^30^ and could be used for treating primary biliary cirrhosis or PBC^31^. Furthermore, a daily dose of 1500 mg for 6 months is well tolerated^32^ and thus appears to have a high therapeutic index. In the context of juvenile diabetes, it has been proposed that TUDCA could be administered on an anticipatory basis to individuals at high risk. Similarly, our results suggest that in treating sepsis, TUDCA could be offered as a preventative therapeutic agent to vulnerable populations i.e. those from the sixth decade of life and above or African-Americans and other non-Caucasian minorities who are consistently at an increased risk of developing sepsis compared to Caucasians.

## Materials and Methods

### Cell lines and culture conditions

Murine RAW264.7 (Kind gift from Dr. Ashley Mansell, Monash University, Melbourne) and MEF cell lines (in-house-derived) were maintained in low glucose DMEM with 10% FCS, 2 mM Glutamine, 1% Pen/Strep at 37 °C and 10% CO_2_. Murine dendritic cell line HAO (Kind gift from Prof. Hans Acha-Orbea. University of Lausanne) was maintained in IMDM with 10% FCS, 2 mM Glutamine, 1% Pen/Strep at 37 °C and 10% CO_2_. The Jurkat human T-cell line (Kind gift from Lorraine O’Reilly, WEHI, Melbourne) was maintained in low glucose DMEM-FMA with 10% FCS, 2 mM Glutamine, 1% Pen/Strep, 250 μM L-asparagine and 50 μM β-Mercaptoethanol at 37 °C with 5% CO_2_ MEFs, Murine RAW264.7 macrophages were seeded at 1 × 10^5^ cells/mL in complete DMEM 10% FCS, 2 mM Glutamine, 1% Pen/Strep prior to activation and incubated overnight at 37 °C 5% CO_2_. The cells were activated with LPS (100 ng/ml) or with LPS plus deoxynivalenol (200 ng/ml) in DMEM, RPMI or FMA (depending on the target cells) for the indicated time points. Conditioned supernatant was harvested 24 hour post activation and filter-sterilized using 0.22 μm filters and either used for experiments directly or kept at −80 °C. THP-1 and U937 cells (human peripheral blood monocyte cell lines, originally from Dr. John Silke, WEHI, Melbourne) were maintained in RPMI-1640 containing 10% FCS at 37 °C and 5% CO_2_.

### Reagents and antibodies

Anti-Bim 3C5 (Enzo Life Sciences, Lausen, Switzerland), anti-CHOP and anti-actin antibodies were described elsewhere^9^. Anti-BiP antibody was a kind gift from Prof. Mary-Jane Gething. Chemical chaperones Genistein (Cat # G6649), Dorsomorphin/compound c (Cat # P5499) and TUDCA (Cat # T0682) were purchased from Sigma-Aldrich (Castle Hill NSW, Australia) and used at the indicated concentrations.

### Cell death assay

For cell death assay, cells were stained with annexin V-FITC or annexin V-AF688 (in house reagents) at the indicated time points at appropriate dilutions followed by PI staining (1 μg/ml, final concentration). For measuring apoptosis in tissue sections at the indicated time points, TUNEL staining was carried out as described previously^33^.

### Protein/RNA analyses

Protein analysis was carried out by Western blot analysis as described before.^9^ RNA analysis was carried out by digital PCR as described before^33^. The oligos used for digital PCR were *XBP1-S* (F): 5′-AAGAACACGCTTGGGAATGG-3′, *XBP1-S* (R): 5′-CTGCACCTGCTGCGGAC-3′; *BIM* (F): 5′-CGACAGTCTCAGGAGGAACC-3′, *BIM* (R): 5′-CCTTCTCCATACCAGACGGA-3′; *PUMA* (F): 5′-GCTGAAGGACTCATGGTGAC-3′ and *PUMA* (R): 5′-CAAAGTGAAGGCGCACTG-3′. XBP splicing analysis by conventional PCR was performed as previously described^34^.

### Animal experiments

All animal experiments were approved by the La Trobe University Animal Ethics Committee (Approval numbers AEC12-66 and AC15-20). Animal experiments were performed in accordance with the guidelines of the Bureau of Animal Welfare, Agriculture Victoria (AG 1005, 2002, revised 2007).

### Cecal slurry injection and the “two-hit” model

The origin and the genotyping protocol for *Bim*^*−/−*^ mice are described previously^22^.

For generating cecal slurry, donor mice were euthanised and cecal contents were suspended in 5% dextrose solution to a final concentration of 250 mg/mL and stored at −80 °C. For experimental induction of sepsis, 8–12 week old *Bim*^*−/−*^ and WT littermate mice of both sexes (C57BL/6) were injected (i.p) with 1 gm/kg cecal slurry. For *in vivo* studies using ER stress inhibitor, mice were injected with TUDCA (500 mg/kg) immediately prior to injection with the cecal slurry or 5% dextrose solution.

For survival studies, animals were subjected to ‘two-hit’ model adopted from Muenzer *et al.*^35^. In brief, C57BL/6 and *Bim*^*−/−*^ mice were injected with PBS (sham) or 0.5 gm/kg cecal slurry with or without TUDCA (500 mg/kg in 0.15 M NaHCO3, pH 7.4) and 3 days post-injection, mice were intra-nasally infected with ∼1.5 × 10^7^ CFU *Pseudomonas aeruginosa* ATC27853. Mice were monitored every 3–6 hours for 10 days during the experiment and animals were culled if their weight dropped <20% bodyweight.

### Cytokine measurement

Serum cytokine levels were measured using BD^TM^ Cytometric Bead Array (Cat # 552364, BD Biosciences, San Diego CA 92121, USA) following manufacturer’s instructions.

### Statistical analysis

GraphPad Prism was used for statistical analyses. P values were calculated either by non-paired two-tailed Student’s *t* - test or by Log-rank test.

## Additional Information

**How to cite this article**: Doerflinger, M. *et al.* Chemical chaperone TUDCA prevents apoptosis and improves survival during polymicrobial sepsis in mice. *Sci. Rep.* **6**, 34702; doi: 10.1038/srep34702 (2016).
